# Precipitation nowcasting with radar data for evaluating multiple horizons using U-Net-based algorithm in Eastern Amazon

**DOI:** 10.1371/journal.pone.0342097

**Published:** 2026-02-05

**Authors:** Rafael Rocha, Douglas Ferreira, Ewerton Oliveira, Helder Arruda, Sergio Viademonte, Ana Paula Paes, Edmir Jesus, Claudia Costa, Vania Franco, Ivan Saraiva, Renata Tedeschi, Antonio Nogueira, Ronnie Alves, Eduardo Carvalho

**Affiliations:** 1 Environmental Intelligence Lab, Vale Institute of Technology, Belém, Pará, Brazil; 2 Nimbus Business, São José dos Campos, São Paulo, Brazil; 3 Centro Gestor e Operacional do Sistema de Proteção da Amazônia, Manaus, Amazonas, Brazil; 4 Association for Information Systems, Atlanta, Georgia, United States of America; Father Muller Charitable Institutions, INDIA; Pacific Northwest National Laboratory, UNITED STATES OF AMERICA

## Abstract

Severe meteorological events are increasingly frequent globally, with intense rainfall significantly impacting well-being, safety, and the economy, including agriculture and mining. Timely emergency alerts are crucial for mitigating losses and preventing fatalities from extreme weather. Precipitation forecasting tools, especially meteorological radars and satellites, are vital due to their high temporal resolution. This study utilizes a U-Net machine learning architecture for spatial-temporal precipitation nowcasting. We evaluate a multi-horizon nowcasting approach using meteorological radar data from the Eastern Amazon, investigating the input data (past horizons) needed for optimal forecast horizons. Our results show that increasing input data beyond 60 minutes degrades performance for short forecast horizon. For short-term forecasts, using 120 minutes of input data instead of 60 minutes resulted in a significant performance loss of 17.60% in RMSE and 7.18% in CSI. These findings identify the optimal input data for accurate nowcasting, enabling safer decision-making during severe weather.

## Introduction

Intense precipitation can substantially impact the well-being, safety, and economic stability of industries and communities worldwide. According to the World Meteorological Organization (WMO) in the “Atlas of Mortality and Economic Losses from Weather, Climate and Water Extremes (1970-2019)”, floods rank among the top disasters (44%) over the last 50 years, with considerable socioeconomic impacts, leading to the loss of 58,700 lives and US $115 billion in economic damages globally [1].

The National Oceanic and Atmospheric Administration (NOAA) classifies a storm as severe when it produces winds over 93 km/h, hail with a diameter over one inch (about 2.54 cm), and/or a tornado. These storms evolve rapidly and are challenging to predict, requiring continuous monitoring for nowcasting. Nowcasting aims to provide real-time (or near real-time) high-resolution (< 1 km) forecasts of local and mesoscale events for short timeframes (up to 2 hours). This process includes the rapid updating (< 1 hour) of observational data on storms, heavy precipitation, strong winds, hail, tornadoes, and visibility (fog), requiring numerical models with the smallest possible temporal (< 1 hour) and spatial (< 1 km) updates [2].

Extreme rainfall events are observed daily in various regions of the country, and their occurrence has been increasing over the years. With continental dimensions, the country experiences meteorological events of all scales, from large to local [3]. These events favor the formation of intense rain clouds, which often reach severe levels and cause damage to society [4]. Tropical clouds can occur individually or in large clusters [5]. These cloud clusters are called convective systems, and the vast majority (˜60%) last less than 9 hours. Typically, they produce lightning over distances of 195 km with a duration of 6 hours, or 330 km with a duration of 27 hours [6]. Particularly in the Amazon region, due to favorable conditions (high temperatures and abundant humidity), deep rain clouds are formed. The interaction of various meteorological systems in the Amazon [7] contributes to the large variability in cloud characteristics across the region. However, in all regions, the potential for severity is recognized, as these clouds often reach altitudes above 16 kilometers [8].

In northern Brazil, the Amazon region has been experiencing increasingly intense and frequent extreme events [9]. Observational studies have identified changes in rainfall patterns in different parts of the Amazon region [10], especially in its eastern portion. These events are directly associated with large-scale patterns modulated by sea surface temperature variability in the equatorial Pacific Ocean, or El Niño–Southern Oscillation (ENSO) phenomenon, and the North and South Tropical Atlantic [11], as well as combined with anomalous conditions in remote regions of the world through teleconnections [12]. In addition to large-scale factors, physiographic characteristics of the region can also contribute to extreme precipitation in the Amazon, such as orography [13].

To ensure decision-makers can effectively implement protective measures to protect lives and mitigate significant economic losses, it’s crucial to predict severe rainfall events in advance. In some industrial sectors such as agriculture [14] and mining [15,16], timely and effective decision-making is essential due to the sudden nature of climate events and the damage they can inflict.

Numerical Weather Prediction (NWP) models are robust and traditional methods used in weather forecasting. NWP models are physic-driven, as predictions are made using mathematical formulations involving atmospheric variables. Despite their effectiveness, NWP models face computational cost constraints due to the time-intensive calculations required for forecasting [17]. Furthermore, NWP models often suffer from the “spin-up” effect, requiring a stabilization period that limits their accuracy for the first few hours of the forecast. In contrast, ML approaches, once trained, allow for rapid inference suitable for real-time applications.

In this context, precipitation nowcasting stands out, aiming to predict the trajectory, duration, and intensity of rainfall events in the very short term (minutes to a few hours) through rainfall estimates based on meteorological radar and satellite data [18,19]. Precipitation nowcasting plays a crucial role in early warning systems for extreme rainfall events in various countries [20,21], especially in issuing early warnings for flash floods [22,23].

In recent years, there has been exponential growth in the search for and use of data-driven methods in weather forecasting. Machine learning (ML) algorithms, in particular, have become prominent for learning from historical meteorological data and using that knowledge to predict future conditions. ML algorithms have shown superior results compared to traditional models like NWP [24]. Additionally, recent methods incorporate multiple variables (such as precipitation, temperature, and wind) from various sensors, and NWP-assimilated data can be used to build datasets for training ML models [25].

ML algorithms have become prominent for learning from historical meteorological data. For instance, ensemble methods combining multiple regressors achieved better rainfall estimates [26]. Similarly, deep learning architectures, such as Multi-Layer Perceptrons (MLP), have shown promising results in integrating passive microwave and infrared data with ground radar products for remote sensing precipitation estimation [27]. Following this trend towards spatiotemporal modeling, a convolutional neural network for precipitation nowcasting was applied to composite reflectivity data in Shanghai, employing a ConvLSTM architecture to transfer information across time steps [28].

Among ML architectures, while Recurrent Neural Networks (RNNs) and ConvLSTMs are designed for sequence modeling, they often incur high computational training costs and can suffer from vanishing gradient issues over long sequences. The U-Net architecture offers a compelling alternative by treating the temporal dimension through channel stacking or 3D convolutions, often achieving faster convergence and comparable performance for immediate nowcasting tasks.

Initially proposed for medical image segmentation tasks [29], the U-Net ML architecture has excelled in state-of-the-art precipitation nowcasting [30–32]. This success is attributed to its autoencoder-like mechanism applied to spatiotemporal forecasting, where a sequence of input images is processed by the U-Net to generate an output sequence of spatially and temporally shifted images (image-to-image translation).

Thus, this work aims to evaluate multi-horizon precipitation nowcasting using data from a weather radar located at eastern Amazon region. The multi-horizon approach investigates the optimal amount of input data needed for accurately predict a given amount of output data, referred to as past and forecast horizons, respectively. This allows us to answer questions like: “To forecast the next 60 minutes of precipitation (forecast horizon), how many previous minutes (past horizons) are needed for an accurate prediction?” For this investigation, the U-Net architecture is used, with each model configured for a specific past and forecast horizon. Additionally, radar data spanning 30, 60, 90, and 120 minutes is tested for both past and forecast horizons.

## Materials and methods

### Study area

The study area is located in the eastern Amazon and includes urbanized zones within the municipalities of Canaã dos Carajás, Curionópolis, and Parauapebas. The region features diverse terrain, ranging from mountain ranges and plateaus to flat areas, with elevations between 200 and 900 m [33]. Its vegetation is also heterogeneous, composed of equatorial forest with trees reaching up to 60 m in height, as well as cerrado formations, grasslands, and canga environments [34]. The area also includes the Carajás National Forest, a federally protected conservation unit that spans the municipalities of Canaã dos Carajás and Parauapebas [35,36].

These municipalities are part of the Itacaiúnas River Basin, which covers approximately 41,500 km^2^ and is subdivided into six main sub-basins: Cateté, Itacaiúnas, Tapirapé, Parauapebas, Vermelho, and Sororó [37]. Canaã dos Carajás and Parauapebas are located within the Parauapebas River sub-basin, the main tributary of the Itacaiúnas River, while Curionópolis lies within the Vermelho River sub-basin [37].

**Fig 1:**
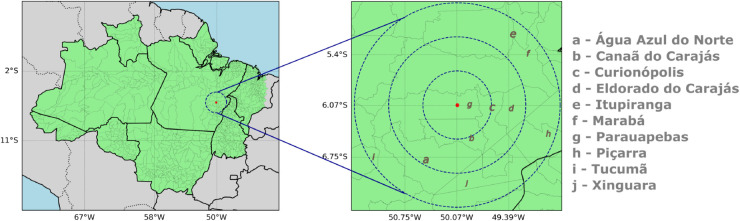
Location of the weather radar (red point), positioned in the eastern Amazon, and some municipalities within its coverage area.

Fig 1 (right) expands the study area, as well as some municipalities within this range. The dashed blue rings indicate distances of 50, 100, and 150 km from the radar’s central location (red point). The closest municipalities to the radar are Parauapebas, Canaã dos Carajás, and Curionópolis, as they fall approximately within the smallest radius circle. Meanwhile, the farthest municipalities, Tucumã, Piçarra, Itupiranga and Marabá, are covered by the largest circle (150 km).

No specific field permits were required for this study as it involved the analysis of remote sensing data obtained from the Vale Institute of Technology’s weather radar.

### Weather radar data

The radar used in this study is an atmospheric monitoring tool manufactured by GAMIC. It is an X-band, dual-polarization radar configured to operate scans with a coverage range of 150 km, a radial beam resolution of 50 m, a pulse repetition frequency (PRF) of 650 Hz, an antenna rotation speed of 2.2 rpm, and a beamwidth of 2.0°. The radar collects samples at every 1° azimuth across 10 elevation angles, ranging from 1.0° (lowest) to 45° (highest) [38]. The radar’s coverage area is shown by the outermost circle (150 km), as depicted on the right in Fig 1.

The weather radar operates with a temporal sampling frequency of 5 minutes, meaning scan volumes are generated at 5-minute intervals. Each volume scan includes information on corrected reflectivity (total, horizontal, and vertical), uncorrected reflectivity (horizontal and vertical), radial velocity (horizontal and vertical), spectral width (horizontal and vertical), differential reflectivity (ZDR), differential phase (PHIDP), specific differential phase (KDP), and the correlation coefficient (RHOHV) [38].

In this work, the variable used for training the predictive models is corrected reflectivity in the dBZ unit, which represents the decibel transformation of Z ($mm^{6}/m^{3}$) and indicates precipitation intensity. The reflectivity used considers the maximum value among the 10 scans obtained by the weather radar, referred to as maximum reflectivity.

Reflectivity is preferred over precipitation in meteorological radar use because it provides a direct measure of the return signal emitted by the radar upon encountering particles in the atmosphere (such as raindrops, snow, hail, or even insects and dust). This return offers an immediate image of the intensity of reflecting particles without relying on accumulation interpretations or indirect processes, as with precipitation [39].

To evaluate reflectivity in terms of precipitation rate (*R*), in mm/h, the Z-R relationship, widely used by meteorologists and originally defined by Marshall and Palmer [40], is considered. The Z-R relationship is a power relationship, meaning any abrupt change in reflectivity values significantly affects the precipitation rate during the conversion. Eq 1 shows the Z-R relationship.

$$Z=200R^{1.6}$$ (1)

While the Z-R relationship allows for the estimation of rainfall rates, this study performs training and evaluation directly on reflectivity (dBZ) values. This approach is chosen to isolate the neural network’s ability to extrapolate spatiotemporal storm patterns from the uncertainties inherent in Z-R parameterization, which can vary significantly depending on the drop size distribution and storm type. Consequently, the results focus on the structural prediction of the meteorological targets.

The reflectivity value of 20 dBZ is commonly used as a minimum threshold to indicate light rain due to several factors: (a) reflectivity around 20 dBZ typically corresponds to a precipitation rate of 0.1 to 1 mm/h, characteristic of light, continuous rain [41]; (b) very low reflectivities (below 20 dBZ) can be caused by noise echoes, small droplets, or even suspended ice particles, which do not necessarily reach the ground as significant precipitation, leading to false positives [39]; (c) meteorological research has found that low-intensity rain begins to be detectable and measurable around this value. Therefore, 20 dBZ is a practical and widely used reference point to signal light rain [42].

### Dataset building

High-resolution data from Climate Prediction Center Morphing (CMORPH) for 2021 were used to select the months with intense precipitation. CMORPH data are high-resolution in both spatial and temporal terms, utilizing precipitation estimates derived exclusively from microwave observations by low-Earth orbit satellites. These features are then transported through spatial propagation information obtained entirely from infrared channel data from geostationary satellites. Days falling within the rainy season, specifically spanning from November 2020 to April 2021, were filtered and used for training precipitation nowcasting models using weather radar data.

**Fig 2:**
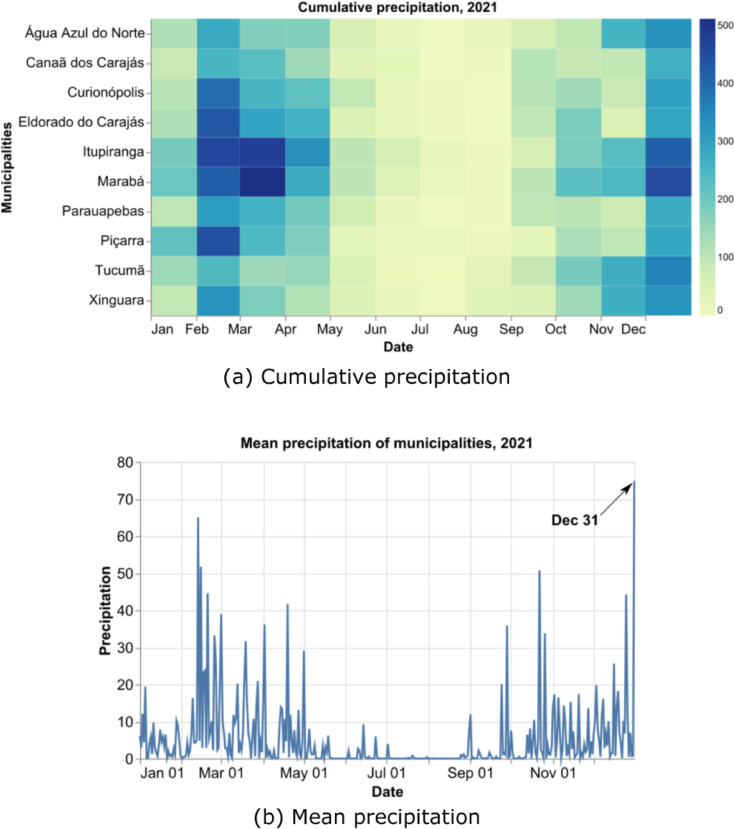
Accumulated precipitation in the municipalities within the weather radar’s coverage area by month (a), and the mean daily precipitation of the municipalities (b), both for the year 2021.

Fig 2a shows the cumulative precipitation of the municipalities within the radar coverage area (Fig 1) by month in 2021, highlighting the rainy season from November to April, represented by high cumulative precipitation values. Fig 2b presents the mean daily precipitation of all the analyzed municipalities throughout 2021, allowing the identification of the rainiest days, with December 31 standing out at 74.87 mm/day.

The rainy season in the region occurs from November to April [43], driven by meteorological systems such as the South Atlantic Convergence Zone (*SACZ*) [44], the Intertropical Convergence Zone (ITCZ) [45], and mesoscale systems like Squall Lines (SL) [46] and Convective Systems, which promote the formation of convective clouds and increase rainfall volume.

**Fig 3:**
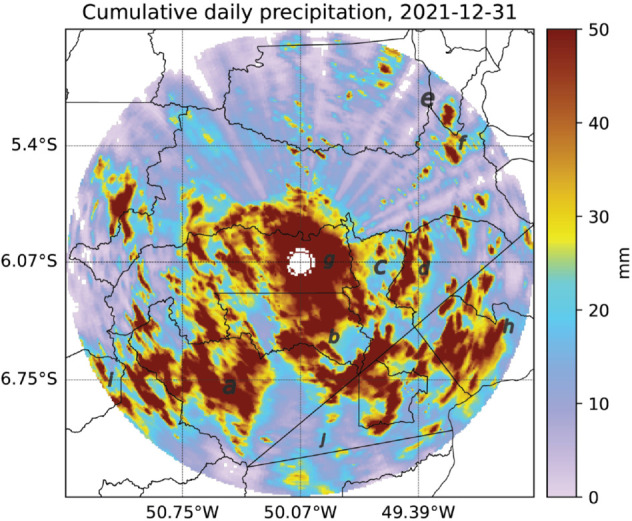
Cumulative daily precipitation on December 31, 2021, obtained from weather radar data. The Z-R relationship was used to estimate precipitation in mm.

The cumulative daily precipitation on December 31, 2021, obtained from the reflectivity variable from the 3 km scan of the weather radar, is shown in Fig 3. High precipitation values can be observed within the radar’s coverage area, indicating the effectiveness of selecting rainy season days using CMORPH data. These days will contribute to building the dataset with weather radar data.

The central region, showing no precipitation in Fig 3, represents the radar’s blind spot. Within a distance of up to 20 km from the radar, scan beams are unavailable at this level, requiring extrapolation from nearby points to fill in the data.

In this context, 28 days from the rainy season of 2021 were selected, generating a dataset of 5,353 reflectivity scans with a resolution of 300 × 300. This dataset was then used to build the input and output sequences for the precipitation nowcasting models to evaluate the multi-horizon approach.

### Multiple horizons approach

The approach used in this work aims to investigate multiple input and output horizons for precipitation nowcasting using weather radar data. In this context, the quantity of input data (horizon) that best fits and performs well in forecasting a specific amount of output data (horizon) is evaluated. In this study, the input data (horizon) is referred to as the past horizon, and the output data (horizon) is defined as the forecast horizon.

**Fig 4:**
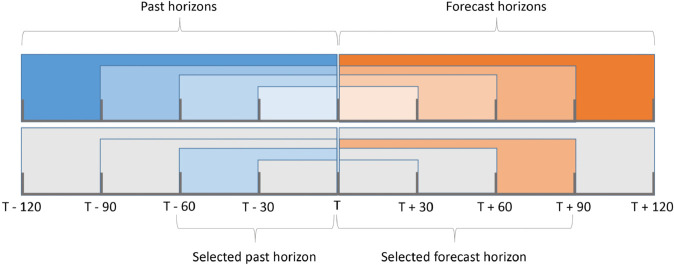
Overview of the multiple-horizon approach for precipitation nowcasting and an example of horizon selection.

Fig 4 presents the approach of multiple past and forecast horizons. The blue-toned windows represent the investigated past horizons, which are used to estimate the forecast horizons shown in orange tones.

In this work, both past and forecast horizons of up to 120 minutes are used, defined as T−120 and T+120, respectively, as shown in Fig 4 (top). Thus, a maximum of 120 previous minutes is investigated to nowcast the next 120 minutes. Furthermore, both horizons vary every 30 minutes, so past and forecast horizons of 30, 60, 90, and 120 minutes are tested.

The selection of a past horizon to estimate a forecast horizon is shown in Fig 4 (bottom). It can be seen that 60 previous minutes, from T−60 to *T* (15:30 to 16:30), are used to nowcast the next 90 minutes, from *T* to T−90 (16:30 to 18:00).

In addition, as shown in Fig 4, the data sequences used for training the precipitation nowcasting models are initially built following fixed 120-minute horizons to ensure that past horizons can be evaluated over the same forecast horizon. Thus, following Fig 4 (bottom), to estimate the 90-minute forecast horizon (orange), it is possible to use the past horizons by selecting only the current past horizon, such as the 60-minute past horizon (blue) in Fig 4 (bottom).

### Precipitation nowcasting model

To create the precipitation nowcasting models for evaluating and comparing the performance of multiple horizons, the U-Net architecture was used. U-Net is a general symmetric U-shaped structure capable of capturing both spatial and temporal information from a sequence of images, dealing with image-to-image translation problems applied to precipitation nowcasting [47].

**Fig 5:**
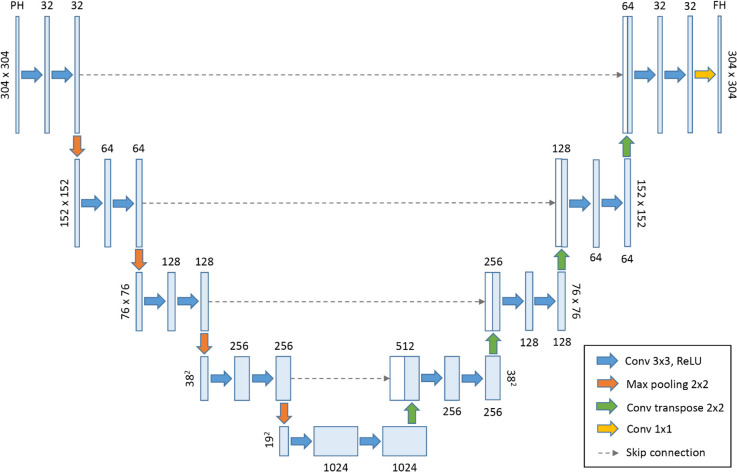
Overview of the U-Net architecture. U-Net is a U-shaped convolutional neural network with an encoder-decoder structure capable of capturing spatio-temporal features.

Fig 5 shows the U-Net architecture used in this work. This model consists of encoder and decoder paths, where the encoder takes a sequence of images defined by the investigated past horizon (*PH*) as input, and the decoder outputs a sequence of images defined by the forecast horizon (*FH*), both with a resolution of 304×304.

The input resolution was adjusted from the original radar grid to 304×304 using zero-padding. This specific dimension was chosen because 304 is divisible by 2^4^(16), which allows the U-Net to perform four distinct down-sampling (max-pooling) operations without resulting in non-integer dimensions. A resolution of 300, for instance, would result in a dimension of 75 at the second level, preventing further symmetrical downsampling and misalignment in the skip connections.

Regarding the network hyperparameters, we adopted standard settings established in precipitation nowcasting literature [29,30]: the Rectified Linear Unit (ReLU) was selected as the activation function to mitigate vanishing gradients, and Batch Normalization was applied to stabilize training. These settings were chosen to establish a robust baseline comparable to state-of-the-art studies, rather than performing an extensive architectural parameter search.

The encoder consists of four blocks of convolutional layers (blue arrows) with a 3×3 kernel size, followed by the ReLU activation function. Between each block, there is a 2×2 max-pooling layer (red arrows) to reduce dimensionality. The decoder blocks have 32, 64, 128, and 256 filters, depending on the U-Net level. The central part of the U-Net is the bottleneck layer, composed of two 3×3 convolutions (blue arrows) with 1024 filters and ReLU. Finally, the decoder consists of four blocks, each composed of a transposed convolutional layer (green arrows) with a 3×3 kernel size to increase the resolution relative to the corresponding level in the U-Net, as well as to reverse the number of filters from the encoder — 256, 128, 64, and 32, respectively. The last block of the decoder, before the output sequence, contains a 1×1 linear convolutional layer (yellow arrow). The skip connections (dashed gray arrows) between levels of the architecture allow for the preservation of small-scale information from the shallower layers by concatenating the decoder features with the corresponding features from the encoder at the same level on the opposite side of the U-Net [48].

### Benchmark models

For comparison with the precipitation nowcasting models built using the U-Net architecture, two baseline models are investigated: semi-Lagrangian extrapolation nowcasting and the Short-Term Ensemble Prediction System (STEPS), both widely used in state-of-the-art precipitation nowcasting.

Nowcasting by extrapolation is commonly used for short-term forecasts, where precipitation patterns are assumed to continue moving at a constant speed without growth or decay in the analyzed variable values, characterizing this approach as deterministic nowcasting [49]. Extrapolation is achieved through the semi-Lagrangian method, a numerical approach used in weather nowcasting models to integrate the equations governing atmospheric motion [50].

STEPS is a stochastic precipitation nowcasting system that combines extrapolation nowcasting with downscaled Numerical Weather Prediction (NWP) [51]. STEPS is a probabilistic nowcasting method as it produces ensemble nowcasts, allowing current forecast uncertainties to be statistically quantified, and it can model growth or decay in the analyzed variable values [52]. For precipitation nowcasting using STEPS, 20 ensembles were considered, representing intermediate complexity, as a higher number of ensembles does not necessarily improve forecast accuracy [53]. The ensemble mean is used for precipitation nowcasting and obtaining results, which is considered the best estimate [54].

Both baseline models use the optical flow approach, which generates motion vectors by estimating the optical flow field from a sequence of data [55]. In this work, optical flow is applied to nowcasting and sequences of weather radar reflectivity scans. The Lucas-Kanade method [56], originally a computer vision technique, is used to estimate optical flow, aiming to track motion patterns between a sequence of radar images for precipitation nowcasting.

### Training parameters

The training of models for evaluating the multi-horizon approach uses the U-Net architecture presented in Fig 5 and detailed in Section. The difference between the models lies in the number of input and output sequences, which are determined by the past and forecast horizons. The past (input) and forecast (output) horizons investigated are for 30, 60, 90, and 120 minutes, where the input and output of the model are defined by sequences of 7, 13, 19, and 25 radar reflectivity scans for the past horizons, and by sequences of 6, 12, 18, and 24 scans for the forecast horizons, respectively.

Although the horizons may represent the same time window, for example, *PH* and *FH* of approximately 60 minutes, the input and output sequences contain different numbers of images (13 and 12, respectively). Thus, the last image in the input sequence represents the most recent observed state from which the model initiates the forecast.

From the selection of 5,353 radar reflectivity scans, 3,981 sequences of input and output images are generated, which are fixed at 25 and 24 images (or past and forecast horizons of 120 minutes). The number of images will be appropriately selected according to the horizons under investigation. The sequences are randomly split using the hold-out method, with 80% allocated for model training and 20% for testing, corresponding to 3,981 and 797 sequences, respectively.

The models are trained over 100 epochs with a batch size of 32. The Adam optimizer was used to minimize the mean squared error (MSE) loss function, which measures the mean squared difference between the target and predicted reflectivity values. Additionally, to ensure compatibility with U-Net training and the proper functioning of the ReLU activation function, only values greater than zero are considered. Values less than zero or NaN (Not a Number), which represents non-reflectivity, are treated as zero.

The Z-score is used to standardize the training and testing sequences to resemble a Gaussian distribution with a mean of zero and a unit standard deviation. Standardization was performed globally using the Z-score method. We computed the mean and standard deviation statistics using the entire training dataset (all input sequences combined), and applied these fixed values to normalize both the training and testing sets. This approach ensures that the physical consistency of reflectivity values, which ranged from 0 to 75 dBZ in our dataset, is preserved relative to the global distribution, rather than normalizing each image individually which could distort intensity scales.

Thus, only four sets of statistics are extracted, as they are defined by the input sequences, which correspond to past horizons (inputs) of 30, 60, 90, and 120 minutes. Therefore, U-Nets trained with a past horizon of 60 minutes are standardized using the same statistics, regardless of the forecast horizon, because the statistics are based solely on the input sequences (past horizon).

The U-Net networks were built using TensorFlow [57] and trained on an NVIDIA Tesla V100 GPU with 32 GB of memory. The pySTEPS library [58] was used for precipitation nowcasting performed by the extrapolation and STEPS approaches.

### Performance evaluation

To quantify the error between target and prediction values, two continuous scores are used: the Root Mean Square Error (RMSE) and the Mean Absolute Error (MAE). RMSE penalizes larger errors more heavily than smaller ones, while MAE provides a more straightforward mean of the absolute differences. The lower the RMSE and MAE scores, the better the model’s performance, indicating smaller differences between target and prediction values. Therefore, the lowest possible score for both scores is 0, and the highest score is undefined, as it depends on the range of values of the variable used. The RMSE and MAE scores are defined by Eqs 2 and 3.

$$\text{RMSE}=\sqrt{\frac{1}{N}\sum_{i=1}^{N}(y_{i}-{\hat{y}}_{i}{)}^{2}}$$ (2)

$$\text{MAE}=\frac{1}{N}\sum_{i=1}^{N}|y_{i}-{\hat{y}}_{i}|$$ (3)

Where *y*_*i*_ and ${\hat{y}}_{i}\in \mathbb{R}$ are the target and predicted values of the reflectivity variable, and N∈ℤ is the number of examples.

To evaluate the models’ ability to predict precipitation events classified as light rain (20 dBZ), where predictions exceed a certain threshold, categorical scores are considered. Thus, the 20 dBZ threshold is used to categorize reflectivity values as “no rain” for values below the threshold and “rain” for events with values above it.

Thus, precipitation nowcasting can be treated as a binary classification task, where the classes are “no rain” and “rain” events. This task is evaluated using scores from the confusion matrix or its equivalent in meteorology, the contingency table. The confusion matrix scores are: true positive (TP), false positive (FP), false negative (FN), and true negative (TN).

Three categorical scores are used to evaluate precipitation nowcasting in terms of binary classification. These are the Critical Success Index (CSI), which measures the fraction of “rain” events (either target or prediction) that were correctly forecasted; the Probability of Detection (POD), which determines the percentage of “rain” events that were accurately predicted; and the Success Ratio (SR), which assesses the proportion of “rain” forecasts that were correctly observed (target). The Eqs 4-6 define these scores.

$$\text{CSI}=\frac{TP}{TP+FN+FP}$$ (4)

$$\text{POD}=\frac{TP}{TP+FN}$$ (5)

$$\text{SR}=\frac{TP}{TP+FP}$$ (6)

It is worth noting that these categorical scores used in meteorology have direct equivalents in computer vision and spatial classification tasks. The CSI score is mathematically equivalent to the Intersection over Union (IoU) score, providing a robust measure of spatial overlap for the binary “rain” class. Similarly, the POD and SR metrics correspond to Recall and Precision, respectively. Therefore, the evaluation provided here encompasses the standard metrics expected for image segmentation tasks.

The TP score indicates the events predicted as “rain” that are indeed “rain”, while FP designates the events predicted as “rain” that are actually “no rain”, FN refers to events predicted as “no rain” that did occur (“rain”), and TN represents events predicted as “no rain” that are indeed “no rain”.

## Results and discussion

**Fig 6:**
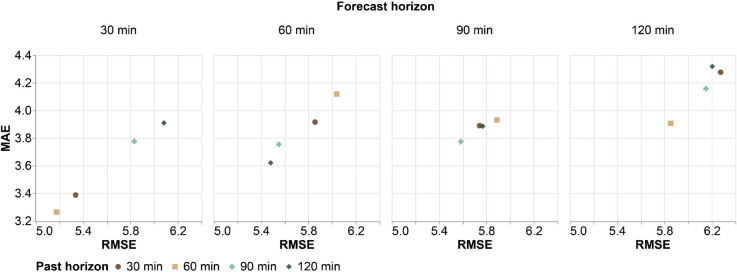
Performance evaluation of multi-horizons through the relationship between RMSE and MAE scores.

A comparison of the multi-horizon models, showing the relationship between the continuous scores RMSE and MAE, is presented in Fig 6. When evaluating the 30-minute forecast horizon, the worst performances are seen with the most distant past horizons (with a larger number of input images), with the 120-minute past horizon standing out, reaching RMSE and MAE values of 6.081 and 3.90, respectively. The best performance past horizon for the 30-minute forecast horizon is the 60-minute past horizon, with RMSE and MAE values of 5.175 and 3.263, respectively.

It can be inferred from the 30-minute forecast horizon results that, in very short-term predictions, the inclusion of older data (90- and 120-minute horizons) introduces outdated or irrelevant storm dynamics, which in turn may hinder the performance of the precipitation nowcasting model.

An opposite trend to the 30-minute forecast horizon results is seen in the analysis of the 60-minute forecast horizon in Fig 6, where the more distant past horizons achieve better performance, with the 120-minute past horizon standing out, achieving the lowest RMSE and MAE values of 5.480 and 3.620, respectively. On the other hand, when evaluating the 90-minute forecast horizon, similar results are observed, with the 90-minute past horizon slightly outperforming the others, achieving RMSE of 5.582 and MAE of 3.774. In contrast, the lowest performance is observed with the 60-minute past horizon, reaching high error values of 5.888 and 3.930 in RMSE and MAE scores, respectively.

In the analysis of the 120-minute forecast horizon results in Fig 6, the best performance is achieved by the 60-minute past horizon, with RMSE and MAE values of 5.852 and 3.906. Additionally, the poorer performance of the 30-minute and 120-minute past horizons highlights that an intermediate past horizon (60 minutes) is more advantageous.

**Fig 7:**
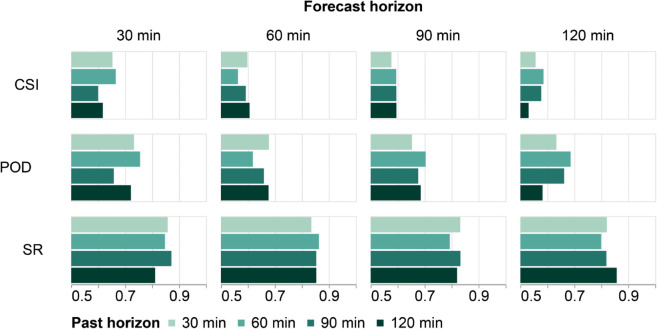
Comparison of multi-horizon performance using categorical scores with the 20 dBZ threshold.

A comparison of multi-horizon performance using the three categorical scores is presented in Fig 7. For the 30-minute forecast horizon, the 60-minute past horizon shows superior performance, particularly for CSI and POD scores, reaching 0.664 and 0.755, respectively. On the other hand, one of the lowest categorical scores is given by the 120-minute past horizon, with a CSI of 0.617 and a POD reaching only 0.720. These results align with those obtained for the same past horizon in the continuous scores.

For the 60-minute forecast horizon, SR scores appear quite similar across past horizons, as seen in Fig 7, although the 60-minute past horizon slightly outperforms with an SR of 0.863. However, when analyzing the other categorical scores, the 60-minute past horizon shows the poorest performance, with CSI and POD scores of 0.562 and 0.617, respectively.

Analyzing the CSI and POD scores for this horizon reveals that the 30 and 120-minute past horizons yield close results, as shown in Fig 7, with the former showing one of the lowest performances and the latter following a trend similar to the continuous scores. The values achieved by the 120-minute past horizon are 0.605 for CSI and 0.676 for POD.

While the 90-minute past horizon achieves the best performance in continuous scores (as shown in the Fig 6), this trend holds only in the SR score for categorical metrics, achieving 0.8324. For the POD score, the 60-minute past horizon stands out, even though it underperformed in continuous scores compared to other models. Finally, for CSI, all horizons greater than 30 minutes perform similarly, with only a 0.04% difference between the 120- and 90-minute past horizons (0.5954 and 0.5950, respectively).

Although the 60-minute past horizon shows lower SR scores for the 120-minute forecast (Fig 7), it achieves the highest CSI and POD scores, reaching 0.585 and 0.686, respectively. These results highlight the effectiveness of the 60-minute past horizon model for forecast values above the threshold (20 dbz) in the 120-minute forecast horizon, as it excels in two categorical scores and all continuous scores (Fig 6).

**Fig 8:**
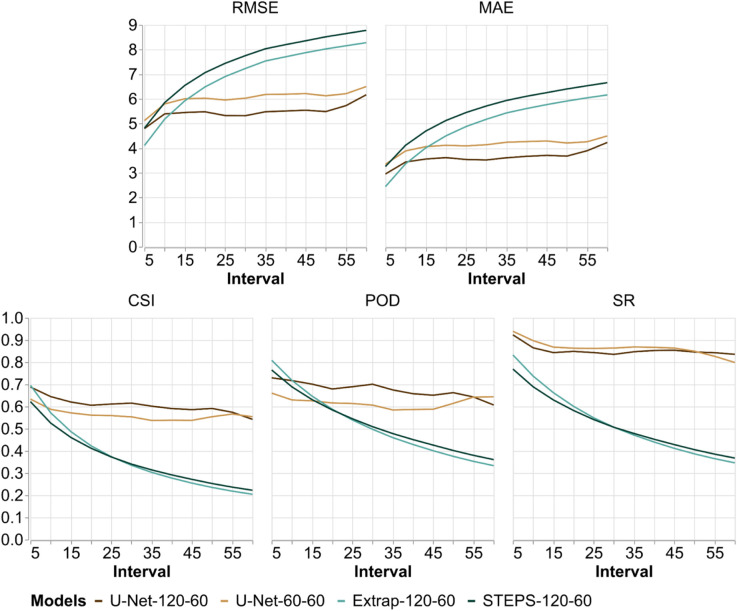
Continuous and categorical scores for each 5-minute interval within the 60-minute forecast horizon. The evaluation includes the best (120-minute past horizon) and worst (60-minute past horizon) models for this forecast horizon, as well as the baseline models Extrapolation and STEPS, both with a 120-minute past horizon.

A comparison based on continuous and categorical scores among four models for the 60-minute forecast horizon across 5-minute time intervals is shown in Fig 8. This includes the past horizons of 120 and 60 minutes (best and worst models) labeled in Fig 8 as U-Net-120-60 and U-Net-60-60, respectively. Additionally, Extrapolation and STEPS models with 120-minute past horizon, noted as Extrapolation-120-60 and STEPS-120-60, are also examined.

Overall, a trend of decreasing performance over time is evident in both score types, with more pronounced degradation in the baseline models, which show lower performance across all scores. When comparing the baseline models, Extrapolation has a slight advantage in categorical scores, while it shows a more marked improvement in continuous scores, with lower errors than STEPS.

For U-Net models, a significant difference is observed between the best (U-Net-120-60) and worst (U-Net-60-60) models in RMSE, MAE, CSI, and POD scores, as shown in Fig 8. The SR score is an exception, with relatively small performance differences but still a slight advantage for U-Net-120-60.

**Fig 9:**
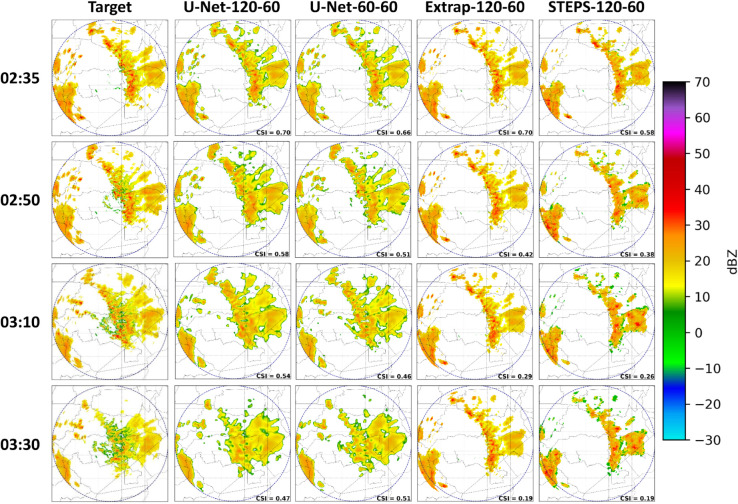
Comparison of precipitation forecasts over various time intervals for models U-Net-120-60, U-Net-60-60, and reference models Extrapolation and STEPS, both with 120-60 horizons.

A comparison of precipitation nowcasts from four models across different time intervals for a 60-minute forecast horizon is presented in Fig 9. These nowcasts represent the weather event on March 19, 2021, from 02:35 to 03:30. Additionally, these nowcasts use 3 km reflectivity scans rather than the maximum reflectivity, which was used to train the U-Nets.

The models visually assessed in Fig 9 are U-Net-120-60, U-Net-60-60, along with the reference models Extrapolation-120-60 and STEPS-120-60. Alongside precipitation nowcasts, CSI score of each nowcast are also included in bottom right of reflectivity map, indicating binary classifications for “no rain” and “rain” events using the 20 dBZ threshold, as previously described.

Fig 9 demonstrates the effectiveness of the four models in capturing the southwestward trajectory of the storm, as indicated by target nowcasts from T+5 (02:35) to T+60 (03:30). For the reference models, a significant decrease in performance is evident as nowcasts extend further from *T*, shown by the decay of CSI score in reflectivity maps, a trend that appears more gradually in the U-Nets. This behavior suggests the reference models forecast a high number of values over 20 dBZ, but these are actually below this threshold.

Conversely, U-Net model forecasts exhibit smoother textures and closer alignment to target nowcasts, as shown by reflectivity scans in Fig 9. In evaluating the CSI score, the U-Net models perform notably better, compared to the reference models. As nowcasts extend from *T*, the U-Net-60-60 model shows a decrease in CSI score relative to U-Net-120-60, which aligns with the superior categorical scores of the U-Net model with a 120-minute past horizon over the time intervals displayed in Fig 8.

Regarding the spatial distribution of errors, the model demonstrates consistent performance across the majority of the radar coverage area, particularly over the central municipalities of interest (Parauapebas and Canaã dos Carajás). A slight degradation in boundary definition is naturally observed at the farthest ranges of the domain (near 150 km) due to radar beam broadening and potential attenuation, but the U-Net architecture effectively maintains storm structure coherence better than the advection-based baselines even in these peripheral regions.

## Conclusion

This study investigates multi-horizons for precipitation nowcasting using weather radar data from eastern Amazonia and the U-Net model. Data from 30, 60, 90, and 120 minutes are analyzed for past and forecast horizons, aiming to determine which past horizon achieves the best performance in predicting a given forecast horizon.

The results for the 30-minute forecast horizon suggest that performance declines as the past horizon extends further back. Notably, comparing the 60 and 120-minute past horizons (best and worst performers) reveals a 17.60% increase in RMSE and a 7.18% decrease in the categorical score CSI.

For the 60-minute forecast horizon, an inverse trend appears, with more distant past horizons yielding better results, as the 60 and 120-minute past horizons demonstrate the worst and best performances, respectively. Between these, there is an approximate 12% decrease in MAE and a 9.45% increase in POD.

An analysis of nowcasts with CSI score confirms the superiority of the 120-minute past horizon in estimating the 60-minute forecast horizon, effectively predicting precipitation values above the 20 dBZ threshold. Additionally, both the 60 and 120-minute past horizon models outperform the reference models.

The results presented in this study demonstrate the effectiveness of the multi-horizon approach in estimating the required amount of input data (referred to as past horizons) to achieve more accurate predictions, effectively capturing the trajectory, intensity, and duration of storms for a given forecast horizon. This enables the selection of the optimal combination of past and forecast horizons, allowing for safe and efficient decision-making up to 120 minutes during intense rainfall events in the eastern Amazon.
